# Effects and Interaction of Meteorological Factors on Pulmonary Tuberculosis in Urumqi, China, 2013–2019

**DOI:** 10.3389/fpubh.2022.951578

**Published:** 2022-07-14

**Authors:** Yanwu Nie, Yaoqin Lu, Chenchen Wang, Zhen Yang, Yahong Sun, Yuxia Zhang, Maozai Tian, Ramziya Rifhat, Liping Zhang

**Affiliations:** ^1^School of Public Health, Xinjiang Medical University, Urumqi, China; ^2^Urumqi Center for Disease Control and Prevention, Urumqi, China; ^3^Center for Disease Control and Prevention of Xinjiang Uygur Autonomous Region, Urumqi, China; ^4^Department of Clinical Nutrition, Urumqi Maternal and Child Health Institute, Urumqi, China; ^5^College of Medical Engineering and Technology, Xinjiang Medical University, Urumqi, China

**Keywords:** pulmonary tuberculosis (PTB), meteorological, interaction, distributed lag non-linear model (DLNM), seasonally

## Abstract

**Background:**

Most existing studies have only investigated the delayed effect of meteorological factors on pulmonary tuberculosis (PTB). However, the effect of extreme climate and the interaction between meteorological factors on PTB has been rarely investigated.

**Methods:**

Newly diagonsed PTB cases and meteorological factors in Urumqi in each week between 2013 and 2019 were collected. The lag-exposure-response relationship between meteorological factors and PTB was analyzed using the distributed lag non-linear model (DLNM). The generalized additive model (GAM) was used to visualize the interaction between meteorological factors. Stratified analysis was used to explore the impact of meteorological factors on PTB in different stratification and RERI, AP and SI were used to quantitatively evaluate the interaction between meteorological factors.

**Results:**

A total of 16,793 newly diagnosed PTB cases were documented in Urumqi, China from 2013 to 2019. The median (interquartile range) temperature, relative humidity, wind speed, and PTB cases were measured as 11.3°C (−5.0–20.5), 57.7% (50.7–64.2), 4.1m/s (3.4–4.7), and 47 (37–56), respectively. The effects of temperature, relative humidity and wind speed on PTB were non-linear, which were found with the “N”-shaped, “L”-shaped, “N”-shaped distribution, respectively. With the median meteorological factor as a reference, extreme low temperature was found to have a protective effect on PTB. However, extreme high temperature, extreme high relative humidity, and extreme high wind speed were found to increase the risk of PTB and peaked at 31.8°C, 83.2%, and 7.6 m/s respectively. According to the existing monitoring data, no obvious interaction between meteorological factors was found, but low temperature and low humidity (RR = 1.149, 95%CI: 1.003–1.315), low temperature and low wind speed (RR = 1.273, 95%CI: 1.146–1.415) were more likely to cause the high incidence of PTB.

**Conclusion:**

Temperature, relative humidity and wind speed were found to play vital roles in PTB incidence with delayed and non-linear effects. Extreme high temperature, extreme high relative humidity, and extreme high wind speed could increase the risk of PTB. Moreover, low temperature and low humidity, low temperature and low wind speed may increase the incidence of PTB.

## Introduction

Tuberculosis (TB) refers to a chronic infectious disease caused by Mycobacterium tuberculosis. Pulmonary tuberculosis (PTB) has been found as the most common type of TB (1). Due to drug resistance and persistence of treatment, TB has been found as one of the main causes of death, ranking first among single infectious diseases. In 2020, about 5.8 million new TB cases and 1.3 million deaths were associated with TB, according to WHO (2). China has a high incidence of TB, with the second highest number of new cases in the world (2). The estimation of TB burden in 2019 suggested that the number of new TB cases in China was 833 thousand, and the incidence rate was 58/100,000. Xinjiang Uygur Autonomous Region is a hotspot for the incidence of TB (3). In 2019, the reported incidence rate was 189.13/100,000 (4), higher than the national average. The incidence of TB in Urumqi, the capital of Xinjiang Uygur Autonomous Region, is not optimistic.

The change of meteorological factors is a hotspot in existing research, as well as one of the biggest challenges facing the world (5). Relevant studies have suggested that with the changes of meteorological factors over the past few years, the suitability of meteorological factors for infectious diseases (e.g., dengue fever, malaria and pathogenic Vibrio) has increased (6). In addition, extreme meteorological factors (e.g., extreme temperature, extreme humidity, and extreme wind speed) significantly affected various infectious diseases (7–10). As a respiratory infectious disease, PTB exhibits seasonal characteristics (11), primarily due to meteorological factors (12, 13). There have been some studies on the relationship between meteorological factors and PTB (14–16), whereas most studies have not investigated the delayed and non-linear dose-response relationship. In addition, Chen et al. adopted the correlation number to explore the effect of extreme temperature on PTB. As revealed by the results, the effect of extreme temperature on PTB was different in different regions (15). However, few people have investigated the effect of extreme humidity and extreme wind speed on PTB. At the same time, the effect of meteorological factors on infectious diseases is often interdependent (17), so it is necessary to investigate the effect of the interaction between meteorological factors on PTB.

The purpose of this study was to analyze the association between meteorological factors and PTB in Urumqi from 2013 to 2019, and to investigate the effect of extreme meteorological factors and the interaction between meteorological factors on PTB. The results are expected to assist in the prevention and control of PTB, thereby reducing the burden of PTB in the future.

## Methods

### Study Sites

Urumqi is located in Northwest China, between 42°45′-44°08′N and 86°37′-88°58′E, taking up an area of 14,216 km^2^. Urumqi has a temperate continental arid climate. It is warm and rainy in summer, and cold and dry in winter. The relative humidity is low, and the temperature difference is large throughout the year. Dust storms often occur in spring and summer (18).

### Data Source

We collected data on meteorological factors of the 8 sites (Supplementary Figure 1), including daily average temperature (°C), daily average relative humidity (%), and daily average wind speed (m/s), between January 1, 2013, and December 31, 2019, from the China Meteorological Information Center (http://data.cma.cn/), as well as the daily new PTB cases during the same period from the Urumqi Center for Disease Control and Prevention. Then, we calculated the weekly averages of PTB cases and meteorological factors for modeling.

### Data Analysis

First, the mean, standard deviation and quantile were used to describe meteorological factors and the number of cases of PTB. Spearman's correlation was adopted to evaluate the correlation between meteorological factors and PTB. The correlation coefficient between temperature and wind speed was found to be large. Accordingly, the variance inflation factor (VIF) and tolerance of meteorological factors were calculated to judge the multicollinearity between variables. Temperature, relative humidity and wind speed were included in the follow-up model.

Second, distributed lag non-linear model (DLNM) was built to estimate the lag and non-linearity of meteorological factors on the risk of PTB. Exposure-response dimension fitting adopted cubic polynomial function without setting nodes; Exposure-lag dimension fitting was modeled by natural cubic splines with 3 degrees of freedom having an internal knot at equally spaced value (19); The degree of freedom of spline function of meteorological factors was set to 3; Since the median incubation period of PTB was 7 weeks (20, 21), while avoiding visit delay and making the model extract more complete information. Thus, the maximum latency was determined as 12 weeks. The number of new PTB cases per week in Urumqi was a small probability event, so this research model used quasi-Poisson to avoid excessive dispersion of data. The time-restricted method (employing simple indicator variables) was used to control the long-term trend and seasonal characteristics (22). The cross-basis functions of temperature, humidity and relative wind speed were built to analyze the lag-exposure-response relationship of meteorological factors. When one factor was included in the function, the other two were set as covariates. Taking the median as the reference value, the influence of meteorological factors on PTB was discussed. The model is expressed as:


(1) $$\begin{array}{l} \log\left[\text{E}\left({\text{Y}}_{\text{t}}\right)\right]=\beta +\text{cb}\left({\text{K}}_{\text{t},12,\beta_{1}}\right)+{\text{s}}_{1}\left(\text{x}\right)+{\text{s}}_{2}\left(\text{z}\right)+\text{strata} \end{array}$$

Where Y_t_ denotes the number of new cases of PTB per week in week t; β represents the intercept of the whole model; cb() represents the cross basis function; K, X, and Z represent meteorological factors; β_1_ denotes the estimated value of the effect of K in a specific lag cycle t; 12 expresses the largest lag order; s() represents the spline function, and the optimal degree of freedom of the spline function was estimated through generalized cross validation; strata was adopted to adjust long-term trends and seasonal characteristics.

The different quantiles of temperature, relative humidity and wind speed (1st, 5th, 95th and 99th) were defined as extreme meteorological factors. On the basis of the above model, taking the median of each meteorological factor as the reference value, the influence of extreme meteorological factors on PTB was discussed.

Subsequently, the generalized additive model (GAM) aimed to explore the interaction of meteorological factors on the risk of PTB. The model is expressed as:


(2) $$\begin{array}{l} \log\left[\text{E}\left({\text{Y}}_{\text{t}}\right)\right]={\text{β}}_{2}+{\text{s}}_{1}\left(\text{k},\text{x}\right)+{\text{s}}_{2}\left(\text{Z}\right)+\text{strata} \end{array}$$

β_2_ denotes intercept; K represents one of the meteorological factors (temperature, relative humidity, and wind speed), X and Z express the other two meteorological factors; s_1_(k, x) denotes the interaction between variables K and X.

Lastly, the effect of the interaction between meteorological factors on PTB was quantitatively explored. With the median as the standard, the meteorological factors were divided into “low” and “high.” For instance, the interaction between temperature and humidity could fall into high temperature and high humidity, high temperature and low humidity, low temperature and high humidity, as well as low temperature and low humidity. One of them was selected as the reference to explore the effect of other factors on PTB. At the same time, the relative excess risk to interaction (RERI), attributable proportion (AP) and synergy index (SI) were used to evaluate the interaction. Interaction is considered to exist only when RERI and AP are not equal to 0 and SI is not equal to 1. The specific calculation formula is as follows:


(3) $$\begin{array}{l} \text{RERI}=\text{R}{\text{R}}_{11}-\text{R}{\text{R}}_{00}-\text{R}{\text{R}}_{01}+1 \end{array}$$

(4) $$\begin{array}{l} \text{AP}=\text{RERI}/\text{R}{\text{R}}_{11} \end{array}$$

(5) $$\begin{array}{l} \text{SI}=\left[\text{R}{\text{R}}_{11}-1\right]/\left[\left(\text{R}{\text{R}}_{10}-1\right)+\left(\text{R}{\text{R}}_{01}-1\right)\right] \end{array}$$

Where RR_00_ (the RR value in the absence of both exposures) is taken as the control group, RR_11_ represents the RR value in the presence of both exposures, and RR_10_ and RR_01_ represent the RR value in the presence of only one of the two exposures.

The impact of meteorological factors on PTB was reported as relative risk (RR) and 95% confidence interval (CI). All statistical analyses were mainly carried out using “dlnm,” “spline” and “mgcv” packages in R software (version 4.0.0). *P* < 0.05 was statistically significant.

## Results

A total of 16793 PTB cases were documented in Urumqi, China from 2013 to 2019. Table 1 lists the characteristics of meteorological factors and PTB. The median (interquartile range) temperature, relative humidity, wind speed, and PTB cases were measured as 11.3°C (-5.0–20.5), 57.7% (50.7–64.2), 4.1m/s (3.4–4.7), and 47 [37-56], respectively.

**Table 1:** Description of meteorological factors and pulmonary tuberculosis (PTB) in Urumqi.

**Variables**	**Mean**	**SD**	**Min**	**P25**	**median**	**P75**	**Max**
PTB (counts/week)	46.1	14.7	4	37	47	56	92
Temperature	8.4	13.4	−21.6	−5.0	11.3	20.5	31.8
Relative humidity	57.7	9.6	29.9	50.7	57.7	64.2	84.0
Wind speed	4.1	0.9	1.9	3.4	4.1	4.7	7.7

*SD, Standard deviation; Min, minimum; P25, the 25th percentile; P75, the 75th percentile; Max, maximum*.

Table 2 lists the spearman's correlation between meteorological factors and PTB. We found that the spearman's correlation coefficient of temperature and wind speed was 0.72, which had a strong correlation. However, VIF and tolerance show that there is no multicollinearity between meteorological factors (Supplementary Table 1). Therefore, we can put multiple meteorological factors into one model. Although the spearman's correlation between relative humidity and PTB is not significant, the impact of meteorological factors on PTB is non-linear and lag, so we still explore the impact of relative humidity on PTB.

**Table 2:** Spearman's correlation between temperature, relative humidity, wind speed, and PTB.

	**PTB (counts/week)**	**Temperature**	**Wind speed**	**Relative humidity**
PTB(counts/week)	1.00	–	–	–
Temperature	0.19*	1.00	–	–
Wind speed	0.17*	0.72*	1.00	–
Relative humidity	0.04	−0.14*	−0.13*	1.00

*P–value < 0.05.

**Figure 1:**
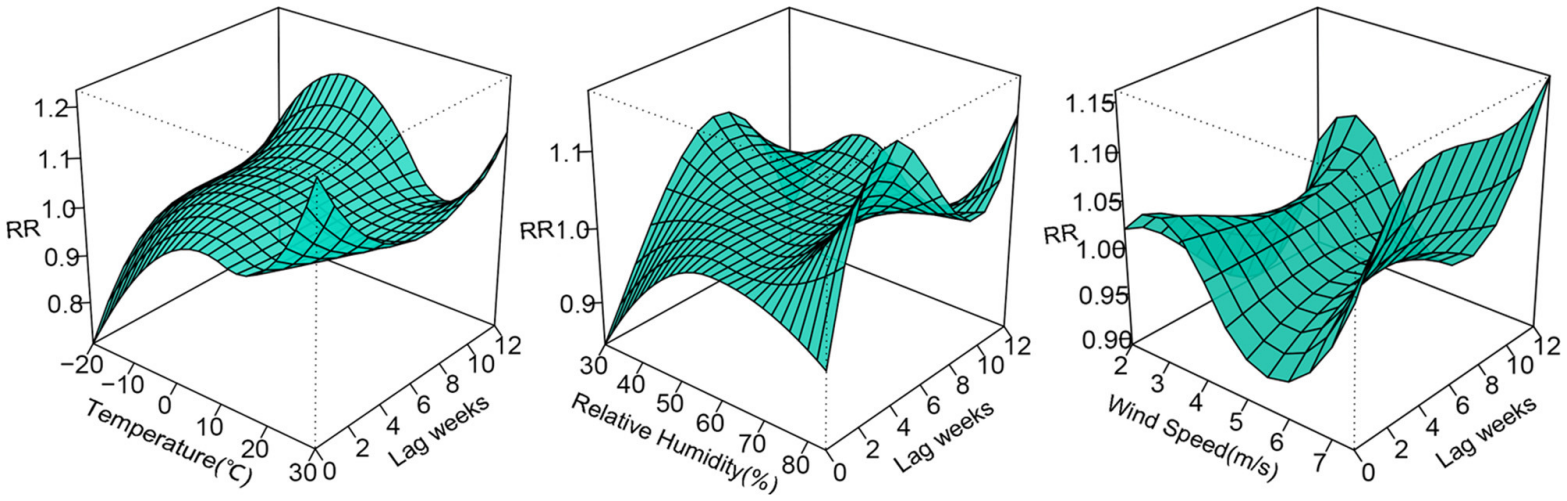
3D plots of temperature, relative humidity, wind speed on PTB at 0–12 lag weeks. The median value was reference.

**Figure 2:**
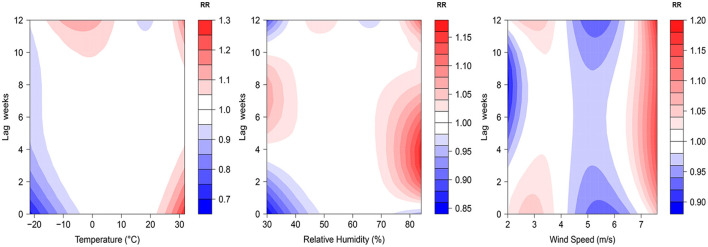
Contour plots of relative risks of meteorological factors on PTB at 0–12 lag weeks. The median value was reference.

The 3D plots show the exposure-lag-response association between meteorological factors and PTB at 0–12 lag weeks (Figure 1). The estimated effects of temperature, relative humidity, wind speed on PTB were non-linear and delayed. Further, the relative risk contour map of meteorological factors on the incidence of PTB at 0–12 lag weeks was drawn (Figure 2). Figure 2 qualitatively shows that higher temperature, humidity and wind speed increased the risk of PTB. In addition, the relative risk (RR) value of PTB increased at week lags 10–12 for the temperature at approximately−10 to 5°C and at week lags 0–3 and 10–12 for the wind speed at approximately 2 to 3.5 m/s.

**Figure 3:**
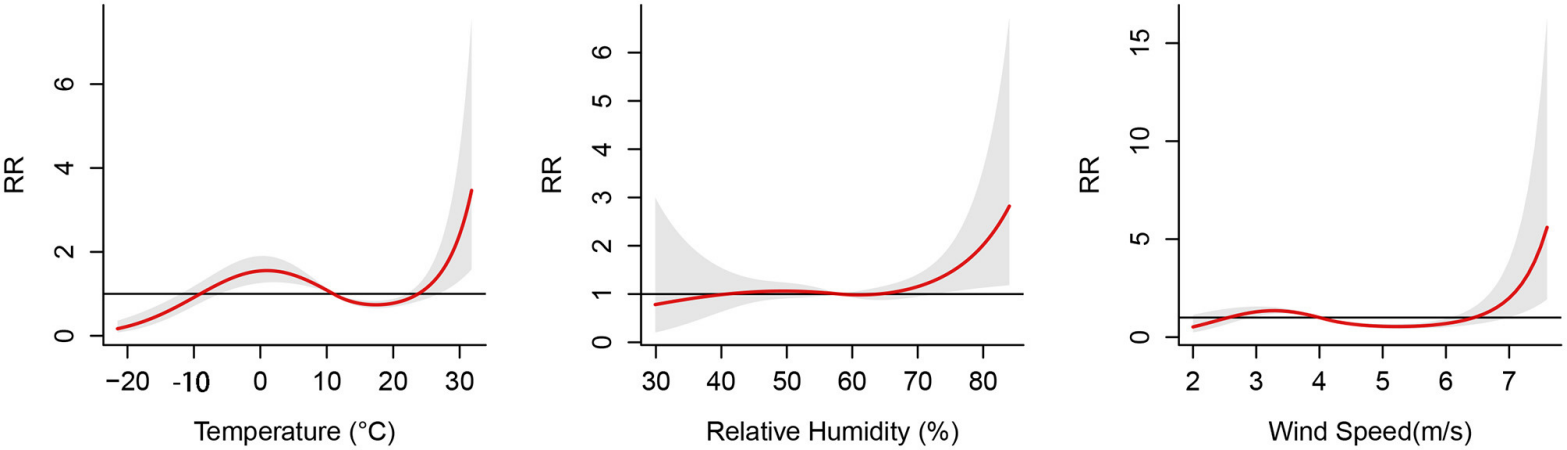
Cumulative effects between meteorological factors and the risk of pulmonary tuberculosis at 0–12 lag weeks. The median value was reference.

Figure 3 illustrates the cumulative effects of temperature, relative humidity and wind speed on the risk of PTB at a lag of 0–12 weeks. The cumulative exposure-response curve of temperature on PTB showed “N”-shaped. The temperature were found with the protective effect at −20 to −9 °C and 11.1 to 23.8 °C, whereas it had statistically significant risk effects at −9.1 to 11°C and 23.8 to 31.8°C. A “L”-shaped distribution was observed between relative humidity and the risk of PTB. When the relative humidity was higher than 64.1 %, the risk of PTB rapidly increased with the increase in the relative humidity. The effect of wind speed on PTN was also approximately “N”-shaped. The wind speed was found with a protective effect at 2 to 2.5 m/s and 4.1 to 6.4 m/s, whereas it had risk effects at 2.6 to 4 m/s and 6.5 to 7.6 m/s.

**Figure 4:**
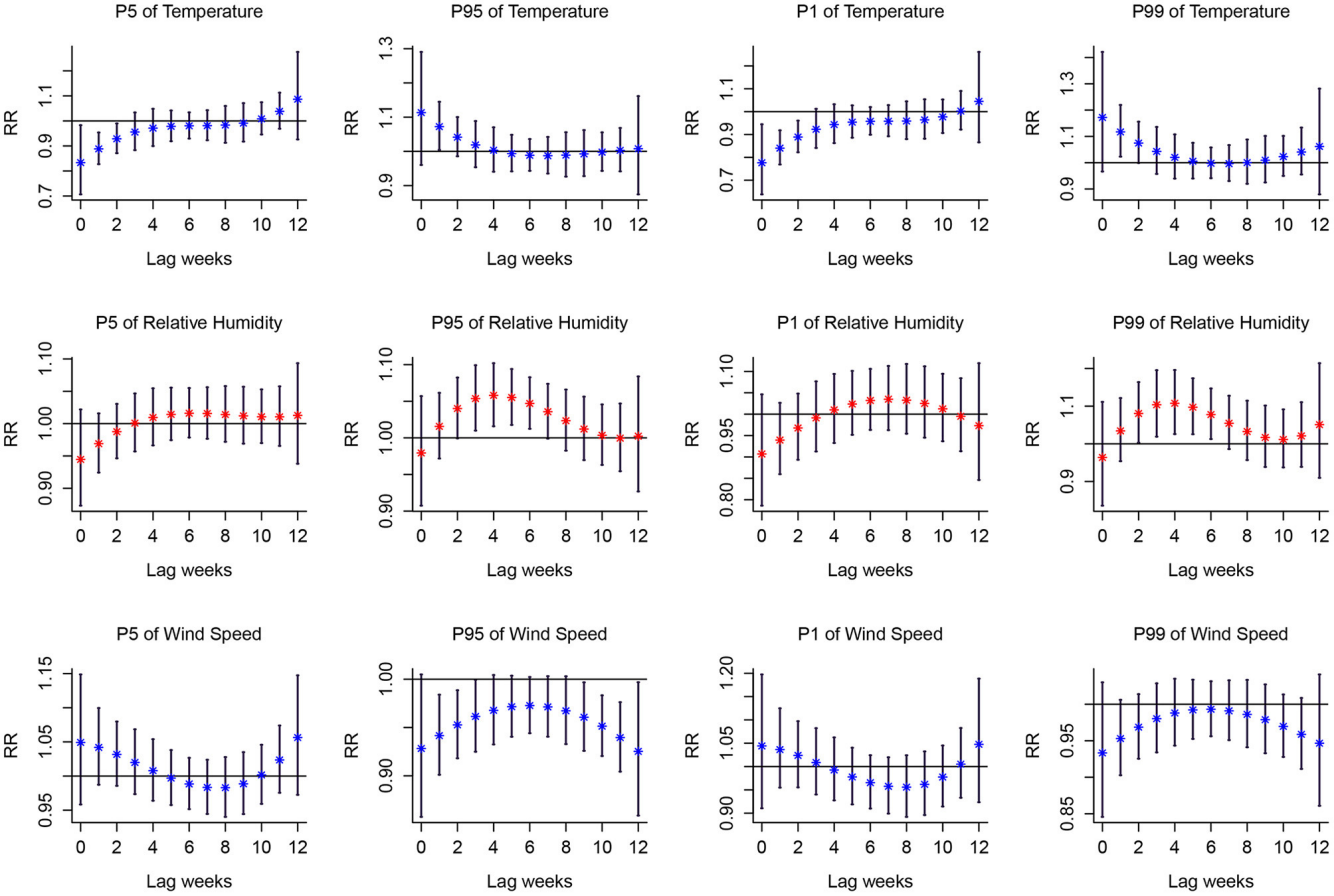
Lag-response curves for P1, P5, P95, P99 of meteorological variables on PTB. P1, the 1th percentile; P5, the 5th percentile; P95, the 95th percentile; P99, the 99th percentile. The median value was reference. Red asterisk and blue asterisk indicate the mean relative risk, and the black vertical line corresponds to 95%CI.

The associations between extreme meteorological factors and the risk of PTB at specific lag weeks are presented in Figure 4. Extreme low temperature was a protective factor of PTB between lag0 to lag2 at the 1th percentile and 5th percentile. Extreme high temperature was a protective factor of PTB between lag1 at the 95th percentile and lag1 to lag2 at the 99th percentile. The effects of extreme high relative humidity peaked at lag4 and was a protective factor of PTB between lag2 to lag7 at the 95th percentile and lag2 to lag6 at the 99th percentile. Wind speed at the 95th percentile was significantly associated with an increased risk of PTB from lag1 to lag3 and lag9 to lag11. However, extreme low relative humidity and extreme low wind speed had no effect on PTB.

Table 3 lists the cumulative effect of extreme meteorological factors (temperature, relative humidity, and wind speed) on PTB with a lag of 0–12 weeks. Extreme low temperature was found to have a protective effect on PTB, and the most significant cumulative effects were reported at the 1st percentile of temperature (RR = 0.416, 95%CI: 0.262–0.659). However, extreme high temperature was found as a risk factor for PTB, with the most significant cumulative effects at the 99th percentile of temperature (RR = 1.675, 95%CI: 1.085–2.587). Extreme high relative humidity increased the incidence rate of PTB in the 99th and 95th percentiles, and it had the most significant cumulative effects at the 99th percentile of relative humidity (RR = 1.905, 95%CI: 1.128–3.216). Compared with the median wind speed, the 95th percentile wind speed was associated with the increased cumulative risk of PTB (RR = 0.547, 95%CI: 0.432–0.692). Extreme low relative humidity and extreme low wind speed did not have any cumulative effect on PTB.

**Table 3:** Cumulative effects of extreme meteorological variables on PTB at 0–12 lag weeks.

	**Temperature (** **°** **C)**		**Relative humidity (%)**		**Wind speed (m/s)**	
**(l0ptr0pt)2-3(l0ptr0pt)4-5 Percentile**	**Value**	**RR (95%CI)**	**Value**	**RR (95%CI)**	**Value**	**RR (95%CI)**
P5	−12.9	0.661 (0.478,0.916)*	42.3	1.015 (0.731,1.408)	2.8	1.182 (0.910,1.537)
P95	25.6	1.220 (0.919,1.619)	74	1.374 (1.026,1.840)*	5.4	0.547 (0.432,0.692)*
P1	−16.3	0.416 (0.262,0.659)*	36.3	0.912 (0.459,1.815)	2.5	0.948 (0.627,1.435)
P99	27.9	1.675 (1.085,2.587)*	79.1	1.905 (1.128,3.216)*	6.4	0.956 (0.632,1.447)

*P–value < 0.05; P1, the 1th percentile; P5, the 5th percentile; P95, the 95th percentile; P99, the 99th percentile; RR, relative risk; CI, confidence interval.

Table 4 lists the largest cumulative effects of temperature, relative humidity, and wind speed on PTB from lag0–1 to lag0–12. The cumulative effect of temperature, relative humidity, and wind speed on PTB peaked at 31.8 °C, 83.2 % and 7.6 m/s, respectively. For temperature, the cumulative lag0–1 to lag0–12 achieved statistical significance; for relative humidity and wind speed, the cumulative lag0–5 to lag 0–12 achieved statistical significance.

**Table 4:** The largest cumulative effects of meteorological factors on PTB by lag period.

	**Temperature (** **°** **C)**		**Relative humidity (%)**		**Wind speed (m/s)**	
**(l0ptr0pt)2-3(l0ptr0pt)4-5 Lag**	**Value**	**RR (95%CI)**	**Value**	**RR (95%CI)**	**Value**	**RR(95%CI)**
0–1	31.8	1.569 (1.046,2.352)*	83.2	1.013 (0.727,1.411)	7.6	1.163 (0.790,1.710)
0–2	31.8	1.808 (1.167,2.800)*	83.2	1.140 (0.771,1.684)	7.6	1.314 (0.820,2.106)
0–3	31.8	1.992 (1.275,3.112)*	83.2	1.319 (0.848,2.051)	7.6	1.509 (0.873,2.610)
0–4	31.8	2.117 (1.339,3.346)*	83.2	1.528 (0.931,2.508)	7.6	1.747 (0.936,3.259)
0–5	31.8	2.191 (1.370,3.504)*	83.2	1.714 (1.009,3.004)*	7.6	2.024(1.010,4.058)*
0–6	31.8	2.231 (1.389,3.584)*	83.2	1.929 (1.075,3.462)*	7.6	2.339(1.096,4.992)*
0–7	31.8	2.260 (1.400,3.648)*	83.2	2.071 (1.118,3.835)*	7.6	2.692(1.194,6.068)*
0–8	31.8	2.304 (1.391,3.815)*	83.2	2.160 (1.129,4.113)*	7.6	3.086(1.298,7.337)*
0–9	31.8	2.393 (1.365,4.194)*	83.2	2.215 (1.114,4.405)*	7.6	3.553(1.406,8.878)*
0–10	31.8	2.564 (1.364,4.824)*	83.2	2.273 (1.097,4.708)*	7.6	4.061 (1.533,10.753)*
0–11	31.8	2.878 (1.441,5.750)*	83.2	2.396 (1.119,5.128)*	7.6	4.719 (1.708,13.041)*
0–12	31.8	3.429 (1.568,7.499)*	*83.2*	2.680 (1.198,5.998)*	*7.6*	5.600 (1.924,16.300)*

*P-value < 0.05; RR, relative risk; CI, confidence interval; The median value was reference.

**Figure 5:**
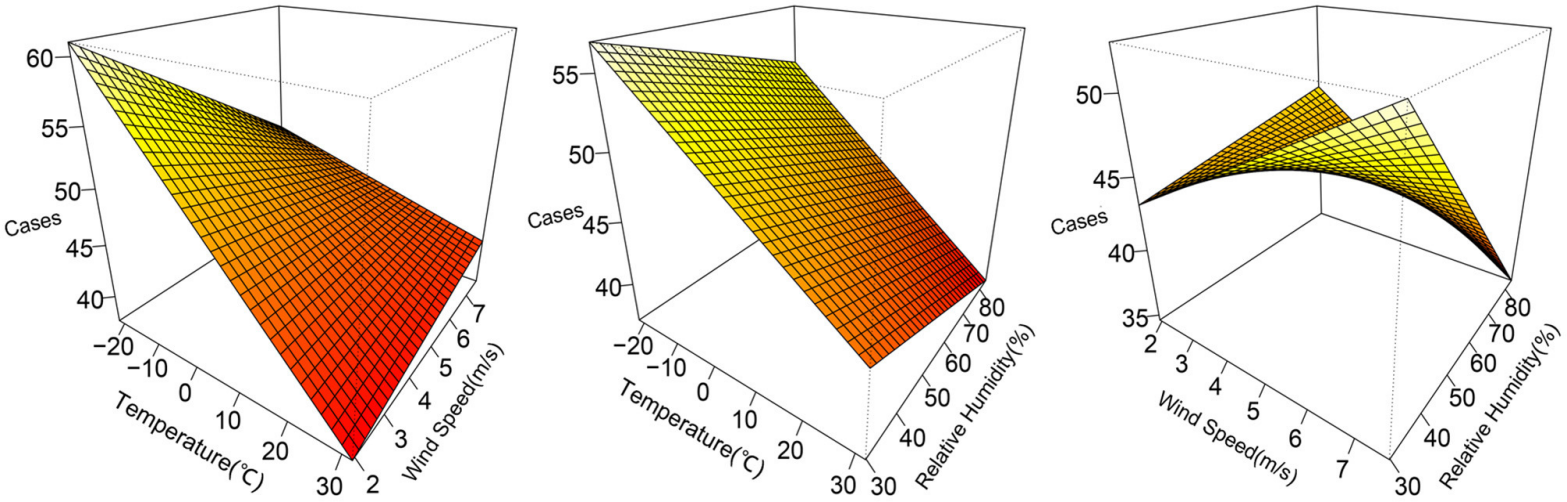
The interaction effect of meteorological variables on PTB after a lag of 12 weeks.

The interaction indicators of additive scale (RERI, AP, SI) showed that there was no obvious interaction between meteorological factors on PTB. However, as depicted in Figure 5, the risk of PTB was higher under low temperature and low wind speed, as well as low temperature and low relative humidity. Further, the meteorological factors were divided in accordance with the median to study the effect of different meteorological stratification on PTB (Table 5). As revealed by the results, compared with high temperature and high relative humidity, the incidence of PTB increased at low temperature and low relative humidity (RR = 1.149, 95%CI: 1.003–1.315). Compared with high temperature and high wind speed, the incidence of PTB increased significantly at low temperature and low wind speed (RR = 1.273, 95%CI: 1.146–1.415). In addition, with high wind speed and high relative humidity as a reference, little difference was found in the effect of PTB whether low wind speed and low relative humidity, or low wind speed and high relative humidity, which indicated that the change of relative humidity may not affect the effect of low wind speed on PTB.

**Table 5:** Effects of different interaction items on the incidence of PTB.

**Interactive item**	**β**	**SE**	**RR (95%CI)**	**RERI (95%CI)**	**AP (95%CI)**	**SI (95%CI)**
Temp and RH				−0.038 (−0.176, 0.100)	−0.033 (−0.153, 0.088)	0.798 (0.363, 1.751)
High temp and high RH	Ref.					
Low temp and low RH	0.139	0.069	1.149 (1.003, 1.315)*			
High temp and low RH	0.054	0.047	1.056 (0.963, 1.157)			
Low temp and high RH	0.123	0.066	1.131 (0.993, 1.287)			
Temp and WS				0.104 (−0.072, 0.280)	0.081 (−0.056, 0.219)	1.611 (0.576, 4.503)
High temp and high WS	Ref.					
Low Temp and low WS	0.242	0.054	1.273 (1.146, 1.415)*			
High temp and low WS	0.087	0.059	1.091 (0.971, 1.223)			
Low temp and high WS	0.076	0.068	1.079 (0.943, 1.232)			
WS and RH				−0.046 (−0.114, 0.021)	−0.042 (−0.102, 0.019)	0.711 (0.457, 1.107)
High WS and high RH	Ref.					
Low WS and low RH	0.108	0.028	1.114 (1.054, 1.178)*			
High WS and low RH	0.044	0.023	1.045 (0.998, 1.094)			
Low WS and high RH	0.109	0.027	1.115 (1.057, 1.177)*			

*P–value < 0.05, Temp, temperature; RH, relative humidity; WS,wind speed; RERI, the relative excess risk to interaction; AP, attributable proportion; SI, synergy index; RR, relative risk; CI, confidence interval. With the median as the standard, the meteorological factors were divided into low and high.

## Discussion

The effect of meteorological factors (temperature, relative humidity, and wind speed) on pulmonary tuberculosis (PTB) in Urumqi was analyzed using the distributed lag non-linear model (DLNM). As revealed by the results, temperature, relative humidity and wind speed played important roles in PTB incidence with effects delayed and non-linear. The extreme high temperature, extreme high relative humidity, extreme high wind speed could increase the risk of PTB. Moreover, low temperature and low humidity, low temperature and low wind speed could increase the incidence of PTB.

The cumulative exposure-response curve of temperature on PTB was “N”-shaped, and the extreme high temperature was found as a risk factor for PTB, similar to the results of Kefyalew Addis Alene et al. (16). After the adjustment for age and gender, an extreme high temperature (29.2°C) was significantly associated with PTB in children (RR = 1.2, 95% CI: 1.01–1.43). Another study from China also suggested that the higher temperature might increase the risk of PTB (23) since the higher temperature would promote the replication of pathogens and enhance the viability of pathogens (24). Furthermore, the air flow at high temperatures was usually very high, thus providing a favorable environment for the spread of PTB (25, 26).

The results confirmed that the effect of relative humidity on PTB showed an “L”-shape. The extremely high relative humidity was found as a risk factor for PTB, consistent with most research results (14, 27). The higher the relative humidity, the stronger the Brownian motion of gas molecules would be, the longer the residence time of Mycobacterium tuberculosis in the air would be, and the greater the possibility of infection with pulmonary tuberculosis would be (14). Moreover, under high humidity, a large aerosol composed of Mycobacterium tuberculosis would be formed, the dose of infectious bacteria entering the human body would increase, the immune system would be overcome and diseases would be spread (28). Furthermore, the contour map of the effect of relative humidity on the incidence rate of PTB indicated that low relative humidity was a risk effect in some lag orders. Thus, the effect of low relative humidity on the risk of PTB cannot be completely excluded.

The relationship between the wind speed, and PTB was also investigated. This study suggested that the lower wind speed was a risk factor of PTB, the higher wind speed was a protective factor of PTB, and extremely high wind speed acted as a risk factor of PTB, well consistent with the results of Cao et al. (23). The low wind speed contributed to the spread of mycobacteria floating in the wind (29), whereas the high wind speed could facilitate air circulation and decrease the incidence rate of PTB (23). The extremely high wind speed might have an effect on the transmission of Mycobacterium tuberculosis, probably because the frequent dust weather in Xinjiang makes Mycobacterium tuberculosis float in the air for a longer time (30).

The results of this study showed that there was no obvious interaction between meteorological factors, but it should also be noted that low temperature and low humidity, low temperature and low wind speed were more likely to lead to the high incidence of PTB. To the best of our knowledge, the effect of the interaction between meteorological factors on PTB has not been explored in existing research. However, an existing study suggested that the transmission of Mycobacterium tuberculosis was significantly associated with the diameter of droplets containing pathogens, and temperature and relative humidity were found as the vital factors for the formation of droplet diameter (31). Droplets with smaller diameter could be formed in the environment of a lower average temperature and lower relative humidity. The smaller the diameter, the slower the landing speed and the longer the residence time in the air would be, so droplets could be easy to be inhaled by susceptible persons (31, 32). In addition, lower relative humidity and lower temperature might increase atmospheric surface dust or polluted particles in winter, and more pathogenic bacteria (e.g., Mycobacterium tuberculosis) may be attached at certain temperature and humidity (33). Moreover, low temperature and low wind speed can also lead to a high incidence of PTB. Low temperature is found in the heating period in Xinjiang (from October to march of the next year), the air pollution is serious (34), the polluted particles may attach more Mycobacterium tuberculosis, and lower wind speed will promote the spread of Mycobacterium tuberculosis.

The influence of meteorological factors on PTB was different in different regions (35), which may be mainly due to the differences in socio-economic levels or analysis methods. The difference of economic level in a region may lead to uneven distribution of social and economic factors, such as food, medical security resources, living conditions, etc (36). Different analysis methods have different requirements for data. Spearman's correlation analysis has no special requirements for the distribution of variables, but the analysis of two time series with long-term trend will lead to biased correlation. The cross-correlation coefficient can deal with the time series with long-term trend, but the influence of meteorological factors on PTB is often non-linear, and the cross-correlation coefficient has limitations in analyzing non-linearity.

The advantages of this study are elucidated below. (1) The effect of extreme humidity, extreme wind speed, and the interaction between meteorological factors on PTB has been rarely studied. (2) Urumqi, Xinjiang is characterized by unique climatic conditions. The temperature, humidity, and wind speed varying greatly within a year were recorded to obtain more comprehensive results. There is sand and dust weather in Xinjiang, which shows certain regional characteristics.

Some limitations should be emphasized. (1) Due to the limitation of data collection, we did not explore the impact of meteorological factors on PTB under different age and gender stratification and the impact of air pollutants on PTB; (2) Using the average method to calculate the personal meteorological factor exposure of 8 meteorological stations may lead to inaccurate personal meteorological exposure; (3) This was a time-series study, which could not determine the causal association between meteorological factors and PTB; (4) The research object of this study was the total number of PTB cases in Urumqi. However, due to the individual differences between groups, we cannot directly extend the results to the individual level, nor to other populations. Further experiments are needed to verify it, so as to avoid the ecological fallacy.

## Conclusion

In brief, the lag and non-linear relationship between meteorological factors and PTB was obtained, and the effect of extreme climatic conditions and meteorological interaction on PTB was also clarified. This study can lay a theoretical basis for developing PTB prevention and control measures. However, the relationship between pollutants and PTB should be explored in depth.

## Data Availability Statement

The raw data supporting the conclusions of this article will be made available by the authors, without undue reservation.

## Author Contributions

YN: methodology, software, data curation, and writing—original draft preparation. YL: methodology, software, visualization, and writing—original draft preparation. CW: methodology, data curation, and writing—original draft preparation. ZY: investigation, software, and visualization. YS: software and visualization. YZ: investigation and data curation. MT: writing—reviewing and editing. RR: writing—reviewing and editing. LZ: conceptualization, supervision, formal analysis, and writing—reviewing and editing. All authors contributed to the article and approved the submitted version.

## Funding

This work was supported by National Natural Science Foundation of China (Grant Nos. 72163033, 72064036, 72174175, and 12101529) and the Tianshan Excellent Youth Project of Xinjiang Uygur Autonomous Region, China (Grant No. 2020Q020).

## Conflict of Interest

The authors declare that the research was conducted in the absence of any commercial or financial relationships that could be construed as a potential conflict of interest.

## Publisher's Note

All claims expressed in this article are solely those of the authors and do not necessarily represent those of their affiliated organizations, or those of the publisher, the editors and the reviewers. Any product that may be evaluated in this article, or claim that may be made by its manufacturer, is not guaranteed or endorsed by the publisher.
