# Supplementary material for: Effects and Interaction of Meteorological Factors on Pulmonary Tuberculosis in Urumqi, China, 2013–2019
Source: Front Public Health. 2022 Jul 14;10:951578. doi: 10.3389/fpubh.2022.951578 (PMC9330012; doi:10.3389/fpubh.2022.951578)
Supplement: Supplementary file 1 [file Data_Sheet_1.pdf]

Supplementary Material

1 Supplementary Figures and Tables

1.1 Supplementary Figures

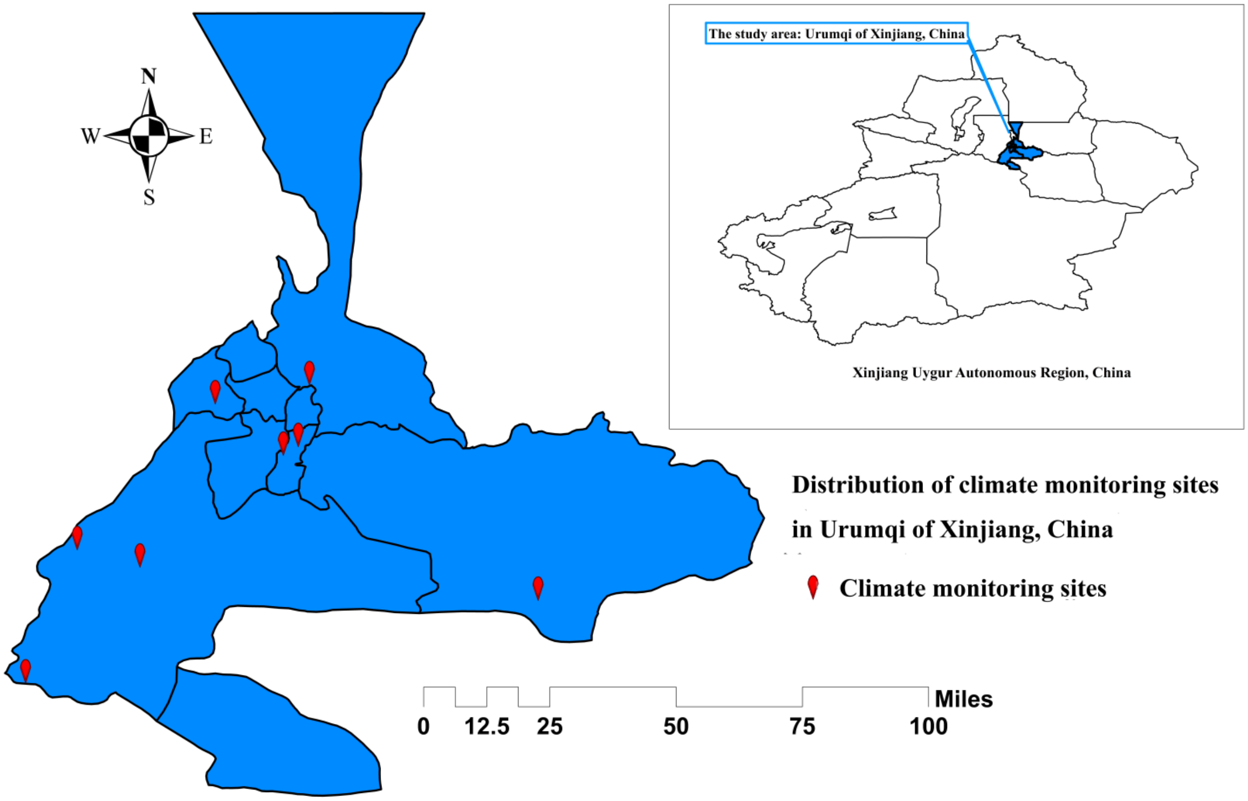

Supplementary Figure 1. The study area and locations of climate stations.

1.2 Supplementary Tables

Supplementary Table 2. Multicollinearity diagnosis among meteorological factors

variable	VIF	tolance
Temperature	1.872	0.534
Wind speed	1.881	0.532
Relative humidity	1.020	0.980
